# Critical Illness Polyneuropathy and Myopathy and Clinical Detection of the Recovery of Consciousness in Severe Acquired Brain Injury Patients with Disorders of Consciousness after Rehabilitation

**DOI:** 10.3390/diagnostics12020516

**Published:** 2022-02-17

**Authors:** Bahia Hakiki, Francesca Cecchi, Silvia Pancani, Anna Maria Romoli, Francesca Draghi, Maenia Scarpino, Raisa Sterpu, Andrea Mannini, Claudio Macchi, Antonello Grippo

**Affiliations:** 1 IRCCS Fondazione Don Carlo Gnocchi, Via di Scandicci 269, 50143 Florence, Italy; bhakiki@dongnocchi.it (B.H.); fcecchi@dongnocchi.it (F.C.); amromoli@dongnocchi.it (A.M.R.); fdraghi@dongnocchi.it (F.D.); maeniascarpino@tiscali.it (M.S.); rsterpu@dongnocchi.it (R.S.); amannini@dongnocchi.it (A.M.); claudio.macchi@unifi.it (C.M.); antonello.grippo@unifi.it (A.G.); 2 Department of Experimental and Clinical Medicine, University of Florence, 50134 Florence, Italy; 3 SODc Neurofisiopatologia, Dipartimento Neuromuscolo-Scheletrico e degli Organi di Senso, AOU Careggi, 50134 Florence, Italy

**Keywords:** severe acquired brain injury, disorders of consciousness, critical illness polyneuropathy and myopathy, Coma Recovery Scale-Revised, neurophysiology, rehabilitation

## Abstract

Background: Disorders of consciousness (DoCs) include unresponsive wakefulness syndrome (UWS) and minimally conscious state (MCS). Critical illness polyneuropathy and myopathy (CIPNM) is frequent in severe acquired brain injuries and impacts functional outcomes at discharge from the intensive rehabilitation unit (IRU). We investigated the prevalence of CIPNM in DoCs and its relationship with the consciousness assessment. Methods: Patients with DoCs were retrospectively selected from the database including patients admitted to the IRU of the IRCCS Don Gnocchi Foundation, Florence, from August 2012 to May 2020. Electroneurography/electromyography was performed at admission. Consciousness was assessed using the Coma Recovery Scale-Revised (CRS-R) at admission and discharge. Patients transitioning from a lower consciousness state to a higher one were classified as improved responsiveness (IR). Results: A total of 177 patients were included (UWS: 81 (45.8%); MCS: 96 (54.2%); 78 (44.1%) women; 67 years (IQR: 20). At admission, 108 (61.0%) patients had CIPNM. At discharge, 117 (66.1%) patients presented an IR. In the multivariate analysis, CRS-R at admission (*p* = 0.006; OR: 1.462) and CIPNM (*p* = 0.039; OR: −1.252) remained significantly associated with IR only for the UWS patients. Conclusions: CIPNM is frequent in DoCs and needs to be considered during the clinical consciousness assessment, especially in patients with UWS.

## 1. Introduction

In recent decades, important advances have been made in emergency medicine and neurosurgical procedures, leading to improved survival of victims of severe acquired brain injuries. After a comatose state, patients may progress to a clinical condition of disorders of consciousness (DoC), which includes unresponsive wakefulness syndrome (UWS), minimally conscious state (MCS), and emergent from the MCS (E-MCS) [1]. These diagnostic categories are defined by the presence and nature (reflex in UWS vs. intentional in MCS) of behavioral responses to multisensorial stimuli. MCS patients can in turn be subcategorized into two distinct entities: on one hand, MCS minus (MCS−), patients showing low-level purposeful behaviors (e.g., visual pursuit, object localization (reaching), and automatic motor response), and MCS plus (MCS+), patients showing a movement to command.

An accurate diagnosis of the consciousness state is crucial since it can influence several aspects of the care pathway, such as treatment and end-of-life decisions, the planning of the rehabilitation path, and the prognosis to be communicated to the patient’s relatives [2]. The Coma Recovery Scale-Revised (CRS-R) [3,4] is the gold standard of consciousness assessment in patients with DoCs [5]. It consists of 23 hierarchically organized items parcellated into six sub-scales assessing different functions with various numbers of hierarchically arranged items. These sub-scales include auditory, visual, motor, oromotor/verbal functions, communication, and arousal scales. The clinical diagnosis of consciousness is based on the presence of the highest level of consciousness assigned in the evaluated sub-scales [3]. Therefore, for each sub-scale, a specific threshold value allows the consciousness stratification into UWS, MCS, or E-MCS [3]. Psychometric studies have widely demonstrated a strong inter-rater and test-retest reliability [3,4] of the CRS-R. However, the scale is subject to inaccuracy, attributable to examiner error and other confounding factors, including comorbidities [6], severe spasticity [7], diffuse pain [8], neuropsychological deficits [9], psychomotor agitation [10], inertia/akinetic mutism [11], iatrogenic effects, presence of tracheostomy [12], and caregiver exclusion during the consciousness evaluation [13], which can lead to misinterpretation of results. In addition, the accuracy of the CRS-R assessment may be affected by extreme motor deficits as reported in previous studies [7].

Critical illness polyneuropathy and myopathy (CIPNM) is the most frequent intensive care unit acquired weakness defined as “clinically detected weakness in critically ill patients in whom there is no plausible etiology other than critical illness” [14]. It often occurs during critical disease [15] and is associated with an increase in intensive care unit morbidity and up to 1 year mortality [16,17]. In the case of cooperative patients, a Medical Research Council scale score < 48 is a clinical criterion for diagnosis of CIPNM [18]. However, the development of clinical diagnostic criteria for CIPNM is hampered by the low reliability of the clinical evaluation of patients with a lack of cooperation, such as patients with DoCs. Therefore, in these conditions, the diagnosis is principally based on the neurophysiological evaluation and electro-diagnostic studies of the peroneal and sural nerves. Among those, the amplitude reduction of the compound muscle action potential of the peroneal nerve has been recognized as the most sensitive and specific neurophysiological parameter [19]. Other electrophysiological tools have been proposed, such as direct muscular stimulation, but a diagnosis of CIPNM subtype can be performed only by biopsy.

In our previous work [20], CIPNM was found in about 50% of patients with severe acquired brain injuries at admission in the intensive rehabilitation unit (IRU). All enrolled patients with severe acquired brain injuries significantly improved during their IRU stay in terms of consciousness state and functional and swallowing abilities, but those with CIPNM achieved lower outcomes. In this study, we aimed to investigate the prevalence of CIPNM only in patients with severe acquired brain injuries admitted with DoC in the IRU and the possible relationship of this peripheral disease with the consciousness state and its evolution between admission and discharge, assessed by the CRS-R.

## 2. Methods

A non-concurrent cohort study was conducted, following STROBE guidelines [21]; the study was performed as an observational retrospective single-site analysis. We followed the principles of the Declaration of Helsinki and the study was approved by the Institutional Ethics Committee (17505_oss).

### 2.1. Participants

In this observational retrospective single-site study, subjects were selected from a database of patients admitted to the IRU of the IRCCS Don Gnocchi Foundation of Florence from August 2012 to May 2020 following severe acquired brain injuries. Written consent was obtained from the legal guardians of all patients, when possible.

Inclusion criteria were admission diagnosis of severe acquired brain injuries, clinical diagnosis of DoC performed by the CRS-R at admission, age 18+ years, and availability of electroneurography/electromyography (ENG/EMG) exam performed within one week of IRU admission. Exclusion criteria were diagnosis of polyneuropathy other than CIPNM (diabetic, alcoholic, and others) and incomplete clinical or instrumental data. All patients were hemodynamically stable, and sedation was withdrawn before IRU admission.

### 2.2. Interdisciplinary Rehabilitation Assessment and Intervention

Within one week of the patient’s sion into the IRU, a team of professionals including a neurologist, internist, physiatrist, physiotherapist, speech therapist, neurophysiopathologist, nurse, and neuropsychologist performed a multidimensional interdisciplinary assessment, including demographics and the clinical data: (a) etiology (traumatic vs. vascular or anoxic); (b) time post-onset (in days); (c) level of consciousness as assessed by CRS-R [4], allowing a consciousness diagnosis in UWS, MCS, or E-MCS; the CRS-R was also reported; and (d) the presence of CIPNM as assessed by the EMG/ENG examination. The CRS-R was assessed by skilled professionals (neurologists and speech therapists).

Based on individual assessments, the individual rehabilitation project was planned by an interdisciplinary team of neurorehabilitation professionals delivering an average of 3 h of specific treatment per day (Table A1, Appendix A). In addition, the pharmacologic interventions were planned according to the patient’s needs. Discharge was planned and carried out upon the decision of the interdisciplinary team, including the patient’s family and caregivers, in agreement with the local health authority, either when the patient reached a plateau or when the patient achieved a functional improvement that allowed home discharge or transfer to a less specialized intensive rehabilitation setting. At discharge, the same professional team repeated the clinical evaluation, including consciousness state based on the CRS-R.

### 2.3. Measures

The CRS-R best scores obtained within the first week following admission and the last week before discharge were used for the patients’ consciousness stratification and retained for the study analyses [22]. At discharge, patients transitioning from UWS to MCS or E-MCS, and from MCS to E-MCS, were classified as patients with IR. Those who remained in their initial state were classified as No-IR patients. 

All included patients underwent a neurophysiological assessment of the four limbs within one week of IRU admission. The ENG/EMG was performed using Medelec Synergy electromyography (Oxford Instrument Medical Ltd., Old Woking, Manor Way, UK). All patients underwent conventional orthodromic motor and antidromic sensory nerve conduction studies on eight motor (axillary, ulnar, common peroneal, and tibial nerves, bilaterally) and four sensory nerves (ulnar and sural nerves, bilaterally). The muscular activity was assessed with concentric needle electrodes at rest and, when possible, during contraction. Sensory nerve action potential, distal motor latencies, F wave, compound muscle action potential, and nerve conduction velocities were registered. Spontaneous activity and, when possible, recruitment and interference patterns were detected bilaterally by needle EMG from the deltoid, abductor digiti minimi, and tibial anterior muscles. For conduction velocities, normal limits were defined as mean ± 2 standard deviations (SD) of normative data of our laboratory [23]. For compound muscle action potential and sensory nerve action potential, the lower limit was set to the 5th percentile derived from the normative data of our laboratory [24]. Diagnosis of CIPNM was made according to amplitude reduction of compound muscle action potential and sensory nerve action potential. Reduction >50% in all four limbs of compound muscle action potentials with or without sensory nerve action potentials amplitude reduction was consistent with CIPNM. Patients with conduction velocities <20% of the lower limit [25] were thought to have a possible diagnosis of polyneuropathy from other causes and were excluded from the analysis as established in the inclusion criteria [23,24].

## 3. Statistical Analysis

Data were analyzed using SPSS software version 27.0 (SPSS Inc., Chicago, IL, USA). The normality of data was assessed with the Shapiro–Wilk test. The categorical variables were summarized as frequencies and percentages, and the continuous ones as median and interquartile range (IQR), as data did not follow a normal distribution. Differences between patients belonging to the UWS and MCS groups were assessed using the Mann–Whitney U test for the continuous variables and the chi-square test or Fisher’s exact test (with Bonferroni adjustment for post hoc comparisons) for the categorical ones. The same tests were used to assess differences between IR and No-IR patients both in the total sample and in the two subgroups (UWS and MCS patients). Statistically significant variables were entered into a multivariate logistic regression model to identify independent predictors of IR. Before inclusion, the Spearman correlation was performed to detect predictors that were highly correlated to avoid multicollinearity. The significance level was set at 0.05 in all analyses.

## 4. Results

**Figure 1:**
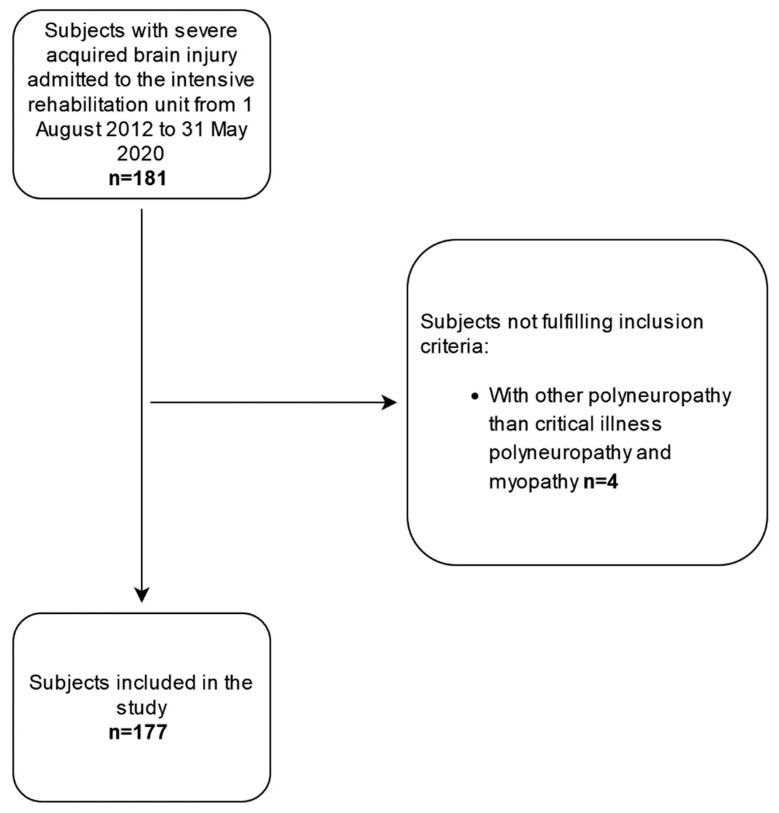
Study flow-chart.

During the study period, 181 patients with DoCs entered the IRU of the IRCCS Don Gnocchi Foundation (Florence, Italy); 4 (2%) were excluded due to polyneuropathy other than CIPNM (Figure 1).

A total of 177 patients (UWS: 81 (45.8%); MCS: 96 (54.2%); women: 78 (44.1%)) were included in the analysis and presented the following characteristics at admission: median age: 67 years (IQR: 55–75), etiology: traumatic (*n* = 44) 24.9%, anoxic (*n* = 25) 14.1%, ischemic (*n* = 25) 14.1%, hemorrhagic (*n* = 70) 39.6%, and others 7.3%: (*n* = 13: metabolic *n* = 2; tumoral *n* = 8; infective *n* = 3), CRS-R median score: 9 (IQR: 5–14); median time post-onset: 43 days (IQR: 32–62). The median length of stay (LOS) was 105 days (IQR: 64–159). One hundred and eight (61.0%) patients were diagnosed with CIPNM at admission. The results of the total sample are summarized in Table 1.

Among the 177 included patients, 117 (66.1%) presented an IR at discharge. When comparing demographics and clinical features of patients who improved their IR at discharge versus those who did not improve, a significantly higher median CRS-R score at admission (11 vs. 5, *p* < 0.001), a higher percentage of MCS diagnosis at admission (64.1% vs. 35.0%, *p* < 0.001), and a lower percentage of CIPNM diagnosis (54.7% vs. 73.3%, *p* = 0.016) were found in patients with IR (Table 1).

We then performed a multivariate regression analysis on the whole sample, introducing all variables that were significantly different between IR and No-IR patients in the univariate analysis, except for the clinical status at admission, which was not included in the multivariate logistic regression because it was highly correlated with the CRS-R total score at admission (ρ = 0.874, *p* < 0.001). The results of the multivariate analysis on the whole sample showed that only a higher CRS-R total score at admission remained associated with a higher probability of IR at discharge (OR 1.169, *p* < 0.001) (Table 2).

Patients were then divided into two groups (UWS and MCS) to account for the influence of consciousness state at admission. The UWS and MCS patients at admission presented significantly different CRS-R scores (*p* < 0.001, Table 3). The percentage of patients with CIPNM diagnosis was higher in the UWS group compared to that in the MCS group (68% vs. 55%), although the difference was not statistically significant (*p* = 0.085). A higher CRS-R score at admission (*p* = 0.004), a shorter time post-onset (*p* = 0.035), and a lower frequency of CIPNM diagnosis (*p* = 0.009) were observed in IR compared to No-IR patients, but only in the UWS group (Table 3), while none of the included features were significantly different between the IR and No-IR patients in the MCS group.

In the UWS group, when variables significantly different between IR and No-IR patients were included in a logistic regression analysis along with age and sex, a higher CRS-R score at admission resulted and was significantly associated with IR at discharge (OR = 1.462, *p* = 0.006; Table 4), while the presence of CIPNM was found to reduce the likelihood of IR (OR = 0.286, *p* = 0.039; Table 4).

## 5. Discussion

In the present study, the prevalence of CIPNM was 61% in patients with DoCs at their admission to the rehabilitative setting. To the best of our knowledge, except for a case study performed on 20 patients [26], this is the first study assessing the prevalence of CIPNM in subjects with DoCs.

This condition continues to be widely underestimated in rehabilitative settings, probably because of the rarity of ENG/EMG examination in both acute and post-acute phases. Consequently, its possible interference both with functional outcomes and the clinical consciousness diagnosis is understated. Our results strongly confirm the recommendation that a systematic neurophysiologic assessment be performed in all patients with DoC, allowing the diagnosis of CIPNM, to improve prognostic assessment and individual rehabilitation project personalization.

We also found that patients with UWS had a lower probability of showing an IR when they suffered from CIPNM. This finding is somewhat puzzling since the relationship between a peripheral disease such as CIPNM and the recovery of consciousness is not immediate. It can be hypothesized that a CIPNM diagnosis is a marker of a higher clinical severity that hinders consciousness recovery independent from the CRS-R score at admission [20]. t was shown that critical illness polyneuropathy, myopathy, or both most commonly develop after acute respiratory distress syndrome, sepsis, systemic inflammatory response syndrome, or multiple organ failure, and that prolonged bed rest, medication, and infections are major risk factors for CIPNM; other major risk factors include long duration of organ dysfunction, parenteral nutrition, vasopressor and catecholamine support, and central neurologic failure (e.g., septic encephalopathy) [27], all of which may affect the probability of recovery of consciousness [28]. However, due to the motor impairment that it causes, the presence of CIPNM might hinder intentional motor responses during the consciousness evaluation, especially in patients with UWS. A previous study by Jorh et al. suggested that the recognition of overt consciousness recovery might be underestimated when severe damage to the motor system that affects motor planning and efferent motor pathways is concomitant, preventing the patient from partially or totally displaying any voluntary responses [29].

Detecting subtle signs of consciousness may have important prognostic, therapeutic, and ethical implications. Several studies have shown that the functional prognosis is affected by the consciousness diagnosis [30,31,32]. Consequently, a correct diagnosis of consciousness might influence pharmacological treatment, decision-making, the design of rehabilitation programs, and family counseling [33]. Knowledge of those confounding factors and clinical situations that may lead to an underestimation of consciousness should be improved, especially when the consciousness diagnosis is exclusively based on clinical observation.

Although the CRS-R has strong evidence of reliability and validity for the assessment of patients with DoC, several studies have highlighted its limitations in some clinical situations [34], identifying some possible confounding factors. To improve CRS-R scoring interpretation, some red flags have been identified to recognize impossible or improbable combinations of specific CRS-R sub-scales that should trigger additional data-quality review to exclude a misdiagnosis of consciousness [35]. Among the relevant confounding factors, quadriplegia was found to generate a warning in the CRS-R scoring due to the combination of zero on the motor sub-scale with the maximum score on the visual or the communication sub-scales [34]. However, since the maximum scoring of the visual or the communication sub-scores is enough to formulate a diagnosis of E-MCS, the presence of quadriplegia does not generate a misdiagnosis of consciousness in patients with a higher level of consciousness. By contrast, it is much more complex to recognize clinical signs of transition from UWS to MCS− [3]. It was shown that five items, including three motor items of the CRS-R assessment, consent to detect 99% of the patients passing from UWS to MCS− [36]. In short, for the nature of the CRS-R, the presence of motor impairment has a higher probability of inducing a consciousness misdiagnosis in the lower states of consciousness (UWS). Therefore, it would be desirable to also include ENG/EMG among the screening examinations in the early rehabilitation phase.

Finally, the use of a multimodal evaluation by combining clinical evaluation and instrumental tools (electroencephalography (EEG), somatosensory-evoked potentials, or functional neuroimaging) is now recommended to improve clinical classification and prognostication of people with DoC [37,38]. Neurophysiological tests, EEGs in particular [39], have proven to be valuable tools to be used alongside the clinical scale to reduce the risk of misdiagnosis in patients with DoC [40]; the presence of CIPNM should be a further reason to recommend such assessments. Furthermore, with a diagnosis of CIPNM, the application of complementary diagnostic tools, such as the motor behavior tool, as proposed for the motor/cognitive dissociation [29], but also the practice of focusing CRS-R motor assessment on facial muscles may help to reduce misdiagnosis.

This study has some limitations that warrant discussion. First, as with all retrospective analyses, we could not control the training background and level of experience of the examiners. However, the agreement between trained investigators who perform the CRS-R evaluation in our center was proven high in an earlier prospective study (kappa coefficient for total scores: 0.827) [30]. Second, data were collected in a single center; therefore, generalization of the obtained results should be made with caution. Finally, the absence of additional information for consciousness diagnosis, either neurophysiological or clinical, prevented us from better elucidating the possible role of severe CIPNM in producing a cognitive motor dissociation and, by this, possibly inducing a misclassification of the level of consciousness assessed by the CRS-R, particularly in patients with UWS. However, the results obtained in this study highlight the need for a deeper and more prospective analysis of this crucial issue; prospective studies combining clinical and instrumental diagnosis of consciousness are required to investigate whether and how CIPNM might impact the consciousness diagnosis in patients with DoC.

## 6. Conclusions

CIPNM is frequent in patients with DoC and has to be taken into account during the clinical assessment of consciousness. CIPNM should be systematically checked at rehabilitation entry in patients with DoC, and because the Medical Research Council scale sum score cannot be obtained in these patients, an ENG/EMG examination should be performed. Complementary tools for the consciousness diagnosis should also be considered in patients with DoC and CIPNM, especially in those classified with UWS, to verify whether severe CIPNM may affect the CRS-R score.

## Figures and Tables

**Table 1:** Characteristics of the study sample and comparison between not-improved responsiveness (No-IR) and improved responsiveness (IR) group.

Variables	Tot (*n* = 177)	No-IR (*n* = 60)	IR (*n* = 117)	*p*-Value
Age	67 (55–75)	68 (54–76)	67 (56–75)	0.709
Sex (F)	78 (44.1%)	28 (46.7%)	50 (42.7%)	0.618
Etiology				0.341
Traumatic	44 (24.9%)	13 (21.7%)	31 (26.5%)	
Anoxic	25 (14.1%)	12 (20.0%)	13 (11.1%)	
Ischemic	25 (14.1%)	6 (10.0%)	19 (16.2%)	
Hemorrhagic	70 (39.6%)	23 (38.3%)	47 (40.2%)	
Other	13 (7.3%)	6 (10.0%)	7 (6.0%)	
Time post-onset	43 (32–62)	46 (35–65)	43 (30–57)	0.148
CRS-R at admission	9 (5–14)	5 (4–10)	11 (7–15)	<0.001
LOS	105 (64–159)	97 (54–146)	111 (69–176)	0.152
Presence of sepsis during IRU stay	54 (30.5%)	20 (33.3%)	34 (29.1%)	0.583
Clinical status at admission				<0.001
UWS	81 (45.8%)	39 (65.0%)	42 (35.9%)	
MCS	96 (54.2%)	21 (35.0%)	75 (64.1%)	
Presence of CIPNM	108 (61.0%)	44 (73.3%)	64 (54.7%)	0.016

Median (interquartile range); frequency (percentage); CRS-R: Coma Recovery Scale-Revised; LOS: length of stay; IRU: intensive rehabilitation unit; UWS: unresponsive wakefulness syndrome; MCS: minimally conscious state; CIPNM: critical illness polyneuropathy and myopathy.

**Table 2:** Multivariate logistic regression analysis for the total study group.

					95% CI for OR	
	B	SE	Sig.	OR	Lower	Upper
Age	−0.006	0.012	0.633	0.994	0.971	1.018
Sex (F)	−0.051	0.357	0.887	0.951	0.472	1.914
CRS-R at admission	0.156	0.038	<0.001	1.169	1.085	1.259
Presence of CIPNM	-0.642	0.381	0.092	0.526	0.250	1.109
Constant	0.152	1.070	0.887	1.164		

Nagelkerke R-square: 0.19. Dependent variable: improved responsiveness. CRS-R: Coma Recovery Scale-Revised; CIPNM: critical illness polyneuropathy and myopathy.

**Table 3:** Characteristics of patients belonging to the unresponsive wakefulness syndrome (UWS) and minimally conscious state (MCS) groups and comparison between those who did not improve responsiveness (No-IR) and those who improved responsiveness (IR) within the groups.

**UWS**	**Tot (*n* = 81)**	**No-IR** **(*n* = 39)**	**IR (*n* = 42)**	***p*** **-Value**
Age	65 (53–74)	65 (53–76)	66 (52–73)	0.744
Sex (F)	37 (45.7%)	18 (46.2%)	19 (45.2%)	0.934
Etiology				0.546
Traumatic	20 (24.7%)	9 (23.1%)	11 (26.2%)	
Anoxic	16 (19.8%)	9 (23.1%)	7 (16.7%)	
Ischemic	11 (13.6%)	4 (10.3%)	7 (16.7%)	
Hemorrhagic	29 (35.8%)	13 (33.3%)	16 (38.1%)	
Other	5 (6.2%)	4 (10.3%)	1 (2.4%)	
CRS-R at admission †	5 (4–6)	4 (3–5)	5 (4–7)	0.004
LOS	95 (57–145)	95 (54–141)	108 (61–157)	0.263
Presence of sepsis during IRU stay	24 (29.6%)	12 (30.8%)	12 (28.6%)	0.884
Time post-onset	42 (30–57)	46 (34–68)	36 (30–50)	0.035
Presence of CIPNM	55 (67.9%)	32 (82.1%)	23 (54.8%)	0.009
**MCS**	**Tot (*n* = 96)**	**NO IR (*n* = 21)**	**IR (*n* = 75)**	***p*** **-Value**
Age	68 (56–76)	71 (55–80)	68 (57–75)	0.380
Sex (F)	41 (42.7%)	10 (47.6%)	31 (41.3%)	0.607
Etiology				0.768
Traumatic	24 (25%)	4 (19%)	20 (26.7%)	
Anoxic	9 (9.4%)	3 (14.3%)	6 (8%)	
Ischemic	14 (14.6%)	2 (9.5%)	12 (16%)	
Hemorrhagic	41 (42.7%)	10 (47.6%)	31 (41.3%)	
Other	8 (8.3%)	2 (9.5%)	6 (8%)	
CRS-R at admission	13.5 (11–16)	12 (10–16)	14 (11–16)	0.117
LOS	107.5 (69–165)	98 (60–161)	111 (75–176)	0.487
Presence of sepsis during IRU stay	30 (31.2%)	8 (38.1%)	22 (29.3%)	0.444
Time post-onset	45.5 (34–64)	45 (38–63)	46 (32–64)	0.797
Presence of CIPNM	53 (55.2%)	12 (57.1%)	41 (54.7%)	0.840

† *p* < 0.05 between UWS and MCS groups. Median (interquartile range); frequency (percentage). UWS: unresponsive wakefulness syndrome; MCS: minimally conscious state; CRS-R: Coma Recovery Scale-Revised; LOS: length of stay; IRU: intensive rehabilitation unit; CIPNM: critical illness polyneuropathy and myopathy.

**Table 4:** Multivariate logistic regression analysis for the UWS group.

					95% CI for OR	
	B	SE	Sig.	OR	Lower	Upper
Age	−0.012	0.019	0.515	0.988	0.953	1.025
Sex (F)	−0.558	0.573	0.330	0.330	0.186	1.760
Time post-onset	-0.015	0.009	0.104	0.985	0.967	1.003
Presence of CIPNM	−1.252	0.607	0.039	0.286	0.087	0.940
CRS at admission	0.380	0.139	0.006	1.462	1.114	1.918
Constant	1.464	1.907	0.443	4.324		

Nagelkerke R square: 0.28. Dependent variable: IR. CRS-R: Coma Recovery Scale-Revised; CIPNM: critical illness polyneuropathy and myopathy.

## Data Availability

The data presented in this study are available on request from the corresponding author.

## Abbreviations

DoC	Disorders of consciousness
UWS	Unresponsive wakefulness syndrome
MCS	Minimally conscious state
CRS-R	Coma Recovery Scale-Revised
CIPNM	Critical illness polyneuropathy and myopathy
IRU	Intensive rehabilitation unit
ENG/EMG	Electroneurography/electromyography
SD	Standard deviations
IR	Improved responsiveness
IQR	Interquartile range
LOS	Length of stay
EEG	Electroencephalography

## Appendix A

**Table A1:** Rehabilitative treatment.

	**LCF = 1–3**	**LCF = 4–6**
Motor Rehabilitation	Individual Neuromotor Physiotherapy To improve surveillance and interaction with the environment To improve or maintain the joint flexibility To prevent spastic hypertone To improve respiratory dynamics by strengthening the inspiratory and expiratory muscles to promote bronchial secretion management To check orthostatic hypotension and neurovegetative reactions during sitting position and verticalization To monitor pain using the nociceptive coma scale To inhibit reflexes and pathological postures	Individual Neuromotor Physiotherapy To promote neurobehavioral alterations control (state of psychomotor agitation, apathy, disinhibition) To improve orientation in time and space To improve focal attention To improve or maintain the joint flexibility To enhance the residual mobility To prevent spastic hypertone To improve respiratory dynamics by strengthening the inspiratory and expiratory muscles to promote tracheobronchial secretion management To check orthostatic hypotension and neurovegetative reactions during sitting position and verticalization To monitor pain using the nociceptive coma scale To prevent musculotendon retraction through passive mobilization To improve balance and posture and postural responses in sitting and orthostatic position To improve trunk control and coordination in simple movements To reduce the Neglet syndrome using the mirror therapy
	**LCF = 1–3**	**LCF = 4–6**
Speach Therapy	To improve surveillance and interaction with the environment To improve swallowing dynamics by promoting oral nutrition To improve the ability to understand verbal/non-verbal messages To improve management of salivary and tracheobronchial secretions To inhibit pathological oral reflexes	To improve swallowing dynamics by promoting oral nutrition with a safe semi-solid/soft diet∙ To improve the ability to understand verbal/non-verbal messages. To improve management of salivary and tracheobronchial secretions To evaluate and manage the weaning path of the tracheostomic cannula To improve the ability to understand verbal/non-verbal messages To improve visuospatial perception To involve the rehabilitation team, the family members and/or caregivers in the observation and stimulation of swallowing and communication functions
Neurocognitive treatment when LCF > 5 and anterograde amnesia resolution		To evaluate the cognitive profile through appropriated cognitive batteries as needed To personify neurocognitive treatment as needed

LCF: Level of cognitive functioning.
